# General Anesthesia: A Probe to Explore Consciousness

**DOI:** 10.3389/fnsys.2019.00036

**Published:** 2019-08-14

**Authors:** Vincent Bonhomme, Cécile Staquet, Javier Montupil, Aline Defresne, Murielle Kirsch, Charlotte Martial, Audrey Vanhaudenhuyse, Camille Chatelle, Stephen Karl Larroque, Federico Raimondo, Athena Demertzi, Olivier Bodart, Steven Laureys, Olivia Gosseries

**Affiliations:** ^1^Anesthesia and Intensive Care Laboratory, GIGA-Consciousness, GIGA Institute, University of Liege, Liege, Belgium; ^2^University Department of Anesthesia and Intensive Care Medicine, Centre Hospitalier Régional de la Citadelle (CHR Citadelle), Liege, Belgium; ^3^Department of Anesthesia and Intensive Care Medicine, Centre Hospitalier Universitaire de Liège (CHU Lièege), Liege, Belgium; ^4^Coma Science Group, GIGA-Consciousness, GIGA Institute, University of Liege, Liege, Belgium; ^5^Sensation & Perception Research Group, GIGA-Consciousness, Department of Algology, GIGA Institute, University of Liege, Centre Hospitalier Universitaire de Liège (CHU Lièege), Liege, Belgium; ^6^Physiology of Cognition Research Lab, GIGA-Consciousness, GIGA Institute, University of Liege, Liege, Belgium

**Keywords:** general anesthesia, consciousness, mechanisms, brain function, brain networks

## Abstract

General anesthesia reversibly alters consciousness, without shutting down the brain globally. Depending on the anesthetic agent and dose, it may produce different consciousness states including a complete absence of subjective experience (unconsciousness), a conscious experience without perception of the environment (disconnected consciousness, like during dreaming), or episodes of oriented consciousness with awareness of the environment (connected consciousness). Each consciousness state may potentially be followed by explicit or implicit memories after the procedure. In this respect, anesthesia can be considered as a proxy to explore consciousness. During the recent years, progress in the exploration of brain function has allowed a better understanding of the neural correlates of consciousness, and of their alterations during anesthesia. Several changes in functional and effective between-region brain connectivity, consciousness network topology, and spatio-temporal dynamics of between-region interactions have been evidenced during anesthesia. Despite a set of effects that are common to many anesthetic agents, it is still uneasy to draw a comprehensive picture of the precise cascades during general anesthesia. Several questions remain unsolved, including the exact identification of the neural substrate of consciousness and its components, the detection of specific consciousness states in unresponsive patients and their associated memory processes, the processing of sensory information during anesthesia, the pharmacodynamic interactions between anesthetic agents, the direction-dependent hysteresis phenomenon during the transitions between consciousness states, the mechanisms of cognitive alterations that follow an anesthetic procedure, the identification of an eventual unitary mechanism of anesthesia-induced alteration of consciousness, the relationship between network effects and the biochemical or sleep-wake cycle targets of anesthetic agents, as well as the vast between-studies variations in dose and administration mode, leading to difficulties in between-studies comparisons. In this narrative review, we draw the picture of the current state of knowledge in anesthesia-induced unconsciousness, from insights gathered on propofol, halogenated vapors, ketamine, dexmedetomidine, benzodiazepines and xenon. We also describe how anesthesia can help understanding consciousness, we develop the above-mentioned unresolved questions, and propose tracks for future research.

## Introduction: General Anesthesia Is More Complex than Simply “Absence of Consciousness”

General anesthesia aims at providing patients with a state where they can tolerate unpleasant and/or noxious interventions, usually during a surgical procedure. Routinely, this involves a cocktail of medications ensuring an alteration of consciousness (pharmacological hypnosis) with absence of awareness of the surrounding environment, explicit recall of undercurrent events, a limitation of the stress response to nociception (anti-nociception), as well as immobility (muscle relaxation). General anesthesia does not shut down the brain globally and does not always produce a complete absence of consciousness. Similarly to disorders of consciousness in patients suffering from a severe brain insult (Aubinet et al., 2018), consciousness may be altered to various degrees, and its alteration may concern different consciousness elements as a function of the equilibrium between the inherent pharmacodynamic properties of anesthetic agents, their concentration in the body, and the intensity of the underlying stimulation by surgery.

Hence, one may not really speak about the depth of anesthetic hypnosis (Bayne et al., 2016), but rather about the presence or absence of one consciousness element or the other. As opposed to concepts that prevailed previously proposing that the brain was simply switched off by anesthesia, it is now clear that subjects may retain several higher-order brain functions until high concentrations of anesthetic agents are attained (Sleigh et al., 2018). If consciousness is defined as reflecting subjective experience/selfhood, or alternatively the sense of being a distinct entity capable of agency, sentience, narrative identity in time, and other higher-order components, anesthesia is capable of suppressing some of these components while keeping others functional (Sleigh et al., 2018). From a more basic and operational point of view, consciousness states that can be observed during general anesthesia are: (i) unconsciousness; (ii) disconnected consciousness; and (iii) connected consciousness (Table 1, Sanders et al., 2012). Unconsciousness can be defined as the inability to achieve any subjective experience and is expected to be the most common anesthetic state. During unconsciousness, reflex motor responses to stimulation may occur, such as arm withdrawal in response to noxious stimulation, but they are not purposeful and voluntary and do not imply a conscious connection with the environment. Disconnected consciousness is characterized by the presence of a mental content, but no conscious perception of the environment. In that case, the mental content can be similar to dreaming (Radek et al., 2018), or more distorted like ketamine-induced psychedelic subjective experiences. Connected consciousness in anesthesia refers to the subjective experience of self, and perception of information from the environment, which may happen in episodes of variable duration, and are not that rare (Sanders et al., 2017; Linassi et al., 2018; Radek et al., 2018). Such episodes can be observed immediately after laryngoscopy and tracheal intubation in approximately 5% of patients (Sanders et al., 2017). Whereas disconnected consciousness refers to internal awareness, connected consciousness refers to both external and internal awareness during anesthesia. External awareness is defined as the perception of environmental sensory stimuli (e.g., auditory, visual, olfactory, or somesthetic), and internal awareness refers to all environmental stimuli-independent thoughts (e.g., inner speech, autobiographical memories, or mind-wandering; Vanhaudenhuyse et al., 2011).

**Table 1:** Possible consciousness states during general anesthesia and their cognitive and mnemonic characteristics.

	Mental content					
Consciousness state	External awareness (perception of environmental sensory stimuli)	Internal awareness (thoughts independent from the environment) and sense of self (agency, sentience, identity, …)	Sensory processing	Purposeful response to command	Explicit memory	Implicit memory
Unconsciousness	No	No	Possible (not accessible from the conscious field)	No	No	Possible
Disconnected consciousness	No	Yes	Yes (not related to external stimulation, e.g., seeing or smelling something during a dream)	No	Possible	Possible
Connected consciousness	Yes	Yes	Yes	Yes	Possible (probably rare)	Possible

Inspired from Vanhaudenhuyse et al. (2011), Sanders et al. (2012) and Sleigh et al. (2018).

The classic method to know in which states patients were during anesthesia is to ask them after recovery, but this delayed assessment misses a lot of events that are not followed by explicit memories. In anesthesia, both disconnected and connected consciousness is rarely followed by explicit recall after the procedure, but the possibility of implicit memories exists. The isolated forearm technique allows assessing connected consciousness “online” during general anesthesia with muscle relaxation, when the patient is unable to manifest consciousness because being paralyzed. This technique consists of isolating the patient’s forearm from the systemic blood circulation through a cuffed upper arm tourniquet, which is inflated before the administration of the neuromuscular blocking agent. In such a setup, the isolated forearm remains non-paralyzed, and the anesthesiologist may ask verbal instructions, such as squeezing the hand and observe the patient’s response (Sanders et al., 2017). The same assessment can be done without the need of a tourniquet when the procedure does not require the use of neuromuscular blocking agents. A debate exists regarding the real incidence and risk factors of connected consciousness episodes during routine anesthesia practice. Although higher incidences have already been reported, the recently published incidence of 5% (and higher in younger patients) immediately after tracheal intubation was claimed to be conservative by the authors, because unclear responses were not counted and because assessment of connected consciousness did not occur during the remaining procedure (Sanders et al., 2017). During experimental studies, it remains important to gather subjective experiences immediately after recovery, to increase the likelihood of catching connectedness during unresponsive periods.

Classical processed electroencephalogram (EEG) indexes of the depth of anesthesia such as the Bispectral Index are not sensitive and specific enough to distinguish between the possible consciousness states of anesthesia (Gao et al., 2018). When asked to cite the consciousness states they would consider acceptable during anesthesia, subjects diversely appreciate one situation or the other. The eventuality of recall or feeling pain are major determinants of their appreciation (Rowley et al., 2017). By reversibly splitting and selectively altering some components of consciousness and selfhood, anesthetic agents are unique tools to explore the associated functional correlates.

Aside from the prerequisite of cortical arousal, which is controlled by sub-cortical sleep-wake cycle regulating systems, the complex phenomena of consciousness and subjective experience/selfhood mainly entail within and between neural networks interactions to generate and integrate information (Tononi, 2004). These activities translate into recordable electrical and metabolic complex signals that can be analyzed using sophisticated techniques. They have been and continue to be used in mostly single anesthetic drug studies in humans, and will ultimately shed light on the specific drug-related functional changes occurring during the different consciousness states of anesthesia, the hysteresis occurring during state transitions in one direction or the other (Kim et al., 2018), sensory processing, explicit or implicit memorization, and the frequently observed functional brain disturbances during the recovery period (Numan et al., 2017). We hereby narratively review the current state of the art in this domain, including studies on propofol, halogenated vapors, ketamine, dexmedetomidine, benzodiazepines and xenon, and underline questions that are not resolved to date.

## Several Ways of Exploring the Effects of Anesthetic Medications on the Brain

**Figure 1:**
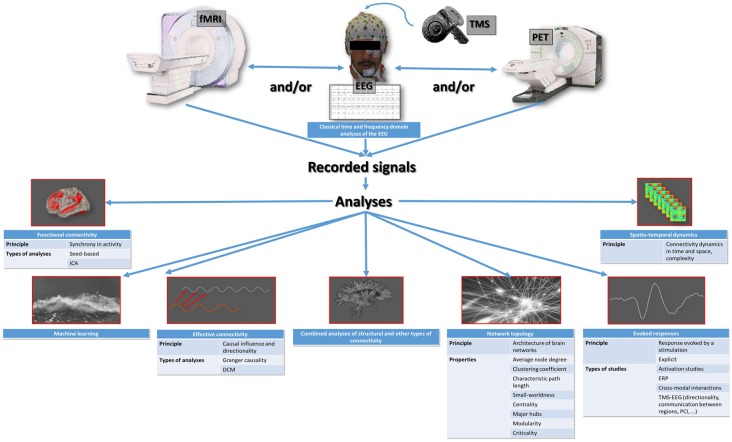
Summary representation of the available types of studies of the functioning brain that are applicable to the exploration of anesthetic brain effects. ICA, independent component analysis; DCM, dynamic causal modeling; ERP, event-related potentials; TMS-EEG, combined electroencephalography and transcranial magnetic stimulation; PCI, perturbational complexity index.

Currently, the exploration of brain function in humans mainly involves EEG recordings, combined with transcranial magnetic stimulation (TMS) or not, and functional brain imaging techniques such as positron emission tomography (PET) and functional magnetic resonance imaging (fMRI). The EEG signal corresponds to the underlying neuronal activities, and the blood oxygen level-dependent (BOLD) signal of fMRI (or the emitted gamma-ray energy in PET) corresponds to changes in regional cerebral blood flow in response to changes in activity. In addition to classical time and frequency domain analyses of the EEG (Marchant et al., 2014) and to classical activation studies of fMRI and PET (Bonhomme et al., 2001), sophisticated analyses can be applied to the recorded signals, with the primary aim to characterize the interactions between different brain regions. These interactions are thought to be the core mechanism of sensory processing and mental content generation (Lee and Mashour, 2018a). The number of possible analysis techniques is high, and each of them addresses a specific aspect of within-brain communication (Figure 1; Mashour and Hudetz, 2018; Staquet et al., 2018). It is therefore important to know exactly what they look at, in order to correctly interpret the results.

The most basic witness of brain communication is between-region synchrony in activity, or functional connectivity. When using fMRI, functional connectivity may be evidenced using hypothesis-based approaches, where brain regions show synchronized (as well as anti-correlated) activity with a predefined seed region (Boveroux et al., 2010; Bonhomme et al., 2016). Hypothesis-free data-driven analyses, for example, Independent Component Analysis (ICA), where signal synchronization is sought globally around the brain (Ribeiro de Paula et al., 2017), can also be used. Effective connectivity refers to the causal influence and directionality of the activity of a brain region on the activity of another region, and may be evidenced using Granger causality (GC) and its derivatives (Nicolaou and Georgiou, 2014), or using dynamic causal modeling (DCM; Gómez et al., 2013; Crone et al., 2017). Another approach, based on graph theory, consists in depicting the architecture or topology of brain networks (Lee and Mashour, 2018a), which are defined as assemblies of nodes (specific brain regions) that are linked by edges (the communication route between two regions). For each network, a set of topological properties can be described, including the average node degree (average number of edges for each node), the clustering coefficient (a measure of segregation, which estimates the isolation of node groups from other groups, a high clustering coefficient corresponds to a high degree of functional specialization), the characteristic path length (average of the minimum number of edges needed to link two nodes, a short path length denotes high integration capacities or efficiency), the participation coefficient (also a measure of integration), the small-worldness (best compromise between functional specialization and efficiency, a small-world network has a high clustering coefficient and short path length), the centrality (index of the central character of a node), the major hubs (most influential nodes, with a high number of edges and a high degree of centrality), the modularity (estimates the separation of a network into modules, which are groups of nodes separated by a hub), and criticality (or metastability, a state between order and disorder resulting from a scale-free organization, allowing the maximum number of possible configurations; Lee et al., 2017). Another mode of investigating brain communication relates to the evolution of the between-region interrelations over time and space, or spatio-temporal dynamics, and their complexity (Untergehrer et al., 2014; Wang et al., 2017; Cavanna et al., 2018; Huang et al., 2018; Muthukumaraswamy and Liley, 2018; Thiery et al., 2018), which is linked to criticality (Barttfeld et al., 2015; Lee et al., 2018). Finally, the analysis of evoked responses to stimulation offers other insights. The oldest studies in that respect were event-related potential EEG studies (Uhl et al., 1980; Plourde and Boylan, 1991), and the fMRI or PET activation studies (Bonhomme et al., 2008). The most recent ones look into how the brain handles sensory information (Lichtner et al., 2018a,b; Nourski et al., 2018) and between-region communication (Darracq et al., 2018a), as well as the directionality of information transfer (Sanders et al., 2018), and sensory cross-modal interactions (Bekinschtein et al., 2009). Some mixed approaches exist, melting one mode of analysis with another, such as those measuring the spatio-temporal complexity of TMS-evoked cortical responses (Casali et al., 2013; Bodart et al., 2017). Most recent emerging techniques use machine learning/decoding approaches through, for example, multivariate pattern analyses, but these have not much been applied to the anesthesia paradigm, yet (Liang et al., 2018). It is also possible to combine structural connectivity analyses (i.e., the exploration of anatomical connections through the white matter), and other types of connectivity analyses such as functional connectivity (Agarwal et al., 2016).

## Current State of the Art—Integrating the Available Data

The process of characterizing changes in brain function during general anesthesia is still ongoing. To date, information has been obtained through studies investigating one single anesthetic agent, mostly during the installing/induction and maintenance phase of sedation, and not all analysis modalities have been applied to each of them. The studies looking at other moments in the anesthesia process (such as the specific aspects of the direction of anesthetic state transitions, recovery as compared to induction of anesthesia, or post-operative delirium), at sensory processing, at memory processes, and at less frequent consciousness states (such as connected consciousness without memorization) are still scarce. A summary of the currently available information follows for each commonly used anesthetic agent (Table 2).

**Table 2:** Summary of the known effects of anesthetic agents on brain function.

	Functional connectivity	Effective connectivity	Topological properties	Evoked responses—sensory processing	Spatio-temporal dynamics
Propofol	Disruption of within- and between-network functional connectivity in large-scale brain networks (particularly fronto-parietal connectivity; Boveroux et al., 2010)	Reduced amplitude and complexity of long-distance cortical communication (Gómez et al., 2013; Sarasso et al., 2015)	Increased local efficiency (parietal regions; Kim et al., 2016; Lee et al., 2017)	Generalized alteration in short-latency evoked electrocorticographic responses to auditory novelty within higher-order cortical areas, outside the auditory cortex (Nourski et al., 2018)	Alteration of dynamics and directionality of effective connectivity (Lee et al., 2009; Untergehrer et al., 2014; Sanders et al., 2018)
	Suppression of the complexity of regions sparsely connected with large-scale brain networks (Pappas et al., 2019)	Disruption of effective connectivity in large-scale brain networks (Lee et al., 2009; Boly et al., 2012; Lee et al., 2018; Untergehrer et al., 2014; Guldenmund et al., 2016; Sanders et al., 2018)	Fewer small-world properties (Barttfeld et al., 2015)	Suppression of long-latency responses to novelty (Nourski et al., 2018)	Reduced complexity and randomness of the electroencephalographic signal (Wang et al., 2017; Darracq et al., 2018b)
	Disruption of thalamo-cortical connectivity within higher-order networks (Boveroux et al., 2010)	Alteration of effective connectivity in lower-order sensory networks (Gómez et al., 2013)	Limitation of connectivity configuration repertoire (Barttfeld et al., 2015; Cavanna et al., 2018)	Reconfiguration of cortical functional connectivity networks involved in nociception, despite activation of spinal cord and cortex by noxious stimulation (Lichtner et al., 2018a,b)	Synchronization of local activity (Huang et al., 2018)
	Preservation of connectivity in lower-order sensory networks (Boveroux et al., 2010)		Traffic of information constrained to inflexible patterns (Mashour, 2018; Uhrig et al., 2018)		Prolongation of long-distance communication timescales (Gómez et al., 2013; Huang et al., 2018)
			Remoteness from criticality, with preserved scale-free organization (Liu et al., 2014; Tagliazucchi et al., 2016; Alonso et al., 2019)		
			Disturbance of posterior parietal hub activity (Lee et al., 2013)		
Halogenated vapors	Breakdown of functional connectivity in higher-order resting-state consciousness networks (Palanca et al., 2015)	Disruption of fronto-parietal anterior to posterior effective connectivity (Lee et al., 2013)	Limitation of connectivity configuration repertoire (Cavanna et al., 2018; Uhrig et al., 2018)	No information	Augmentation of temporal persistence in neuronal oscillation amplitude (Thiery et al., 2018)
	Disruption of thalamo-cortical connectivity within higher-order networks (Palanca et al., 2015)		Remoteness from criticality (Lee et al., 2018)		Disruption of intermediate strength spatio-temporal patterns of functional connectivity within and between consciousness networks (Kafashan et al., 2016)
	Preservation of connectivity in lower-order sensory networks (Ranft et al., 2016)				Preservation of higher strength spatio-temporal patterns within networks (Kafashan et al., 2016)
Ketamine	Global increase in functional connectivity, with network reorganization (Driesen et al., 2013b)	Disruption of fronto-parietal anterior to posterior effective connectivity (Lee et al., 2013; Vlisides et al., 2017)	No information	TMS-evoked communication complexity close to the waking state (Sarasso et al., 2015)	No information
	Disruption of functional connectivity in all higher-order consciousness networks but not in the executive control network (Bonhomme et al., 2016)	Reduced alpha power in the precuneus and temporo-parietal junction (possibly related to disconnected consciousness; Vlisides et al., 2018; Darracq et al., 2018a)			
	Preservation of functional connectivity in sensory networks (Bonhomme et al., 2016)				
	Long-term effect on the interactions between the default mode network and networks involved in depression? and restoration of the abnormal connectivity of depressed patients (Li et al., 2018; Vutskits, 2018)				
	Transient effect on working memory network (Driesen et al., 2013a)				
Dexmedetomidine	Reduced within-network and thalamic connectivity in higher-order consciousness networks (Guldenmund et al., 2017)	No information	Reduced local and global large-scale network efficiency (Hashmi et al., 2017)	No information	No information
	Preservation of lower-order sensory networks functional connectivity (Guldenmund et al., 2017)		Reduced large-scale network connectivity strength (Hashmi et al., 2017)		
	Better preservation of functional connectivity between thalamus, medial anterior cingulate cortex, and mesopontine area as compared to sleep and propofol unresponsiveness (Guldenmund et al., 2017)		No impairment in node degree (Hashmi et al., 2017)		
Benzodiazepines	Disruption of higher-order consciousness networks (Greicius et al., 2008; Liang et al., 2018)	Disruption of effective connectivity in large-scale brain networks (Greicius et al., 2008; Ferrarelli et al., 2010; Liang et al., 2018)	No information	Reduced auditory cortex activation by sounds(Frolich et al., 2017)	No information
	Preservation of lower-order sensory networks (Frolich et al., 2017)			Reduced duration and propagation of evoked TMS cortical response (Ferrarelli et al., 2010)	
Xenon	No information	No information	Remoteness from criticality (Colombo et al., 2019)	TMS-evoked high amplitude slow waves with low complexity (Sarasso et al., 2015)	Slowing down and smoothing of the temporal profile of the EEG signal (Colombo et al., 2019)

### Propofol

Thanks to its safety and ease of use, the γ-amino-butyric acid (GABA) neurotransmission promoting agent propofol has been the most widely studied anesthetic agent with respect to brain mechanisms in primates and healthy humans. Years after the pioneering works of Fiset and Alkire, who were the first to demonstrate region-specific and dose-dependent effect of propofol on brain activity (Alkire et al., 1995; Fiset et al., 1999), it came out that this agent diminishes the randomness of the spontaneous and evoked EEG signal (Wang et al., 2017; Darracq et al., 2018b), alters long-distance cortical communication (Gómez et al., 2013) and reduces its complexity (Sarasso et al., 2015), disrupts within- and between-network functional connectivity in large-scale brain networks that sustain consciousness (Boveroux et al., 2010), and particularly functional fronto-parietal connectivity. Recent findings also suggest the suppression of the complexity of regions sparsely connected with large-scale brain networks as a mechanism of propofol-induced alteration in oriented reactivity to stimulation (Pappas et al., 2019). Effective connectivity, its dynamics, and its directionality are altered by propofol (Lee et al., 2009, 2013; Boly et al., 2012; Untergehrer et al., 2014; Guldenmund et al., 2016; Sanders et al., 2018). Contrarily, propofol relatively preserves lower-order sensory networks connectivity but impedes the effective connectivity within them (Gómez et al., 2013). When looking at network topological properties, propofol-induced unresponsiveness is associated with increased local efficiency, particularly in parietal regions (Lee et al., 2017), reduced global efficiency (Kim et al., 2016), disturbance of the posterior parietal hub activity (Lee et al., 2013), fewer small-world properties (Barttfeld et al., 2015), a limitation of the repertoire of possible connectivity configurations (Barttfeld et al., 2015; Cavanna et al., 2018) by constraining the traffic of information to inflexible patterns (Mashour, 2018; Uhrig et al., 2018), and remoteness from criticality (Tagliazucchi et al., 2016) with preserved scale-free organization of networks (preservation of node size and node degree distribution; Liu et al., 2014). Under propofol, the dynamics of within-brain interactions become more stable (Alonso et al., 2019). Propofol also synchronizes local activity and prolongs the timescales of long-distance communications (Huang et al., 2018). The consequences of propofol infusion on sensory processing involve a generalized alteration in short-latency evoked EEG responses to auditory novelty (which are thought to reflect pre-attentive processing) within higher-order cortical areas, but outside the auditory cortex. Long-latency responses to novelty (which may reflect conscious processing) are markedly suppressed by propofol in all regions (Nourski et al., 2018). Noxious information still reaches the cortex through the spinal cord, even under high propofol concentrations (Lichtner et al., 2018a), and in the presence of remifentanil (Lichtner et al., 2018b), but the cortical functional connectivity networks usually involved in nociception are reconfigured (Lichtner et al., 2018a).

### Halogenated Vapors

Similarly to propofol, the inhaled halogenated vapors such as sevoflurane have GABAergic properties in addition to other biochemical targets such as potassium channels (Bonhomme et al., 2011). They break down functional connectivity in higher-order resting-state large-scale networks such as the default-mode network, and the salience network, as well as the thalamo-cortical connectivity within them (Palanca et al., 2015), with a preservation of connectivity within sensory networks (Ranft et al., 2016). The fronto-parietal anterior to posterior effective connectivity is also reduced by sevoflurane at anesthetic doses (Lee et al., 2013; Ranft et al., 2016). Halogenates put networks aside from criticality (Lee et al., 2018), and limit the repertoire of possible network configurations (Cavanna et al., 2018; Uhrig et al., 2018), possibly through an augmentation of temporal persistence in neuronal oscillation amplitude (Thiery et al., 2018). The intermediate strength spatio-temporal patterns of functional connectivity are disrupted within and between networks, while higher strength patterns are preserved within networks (Kafashan et al., 2016), and fewer transitions in between-networks connectivity patterns occur (Golkowski et al., 2019).

### Ketamine

Among anesthetic agents, ketamine can be considered as the black sheep, because it induces distinct behavioral and functional changes as compared to other agents. The N-methyl-D-aspartate (NMDA) glutamate receptor antagonist ketamine produces a dissociative state with disconnected consciousness, through an isolation of the individual from the environment, and the emergence of intense dreaming with hallucinations and distorted self-perceptions. Ketamine increases functional connectivity globally (Driesen et al., 2013b). This occurs through a reconfiguration of between-region communication that becomes ineffective for some networks. Indeed, ketamine disrupts functional connectivity in all higher-order networks but the executive control network (Bonhomme et al., 2016). Also, connectivity is further preserved in sensory networks. A disruption of the fronto-parietal effective connectivity, and particularly the connectivity going from the anterior to the posterior part of the brain, has been observed during ketamine anesthesia (Lee et al., 2013; Vlisides et al., 2017). It induces a degree of TMS-evoked communication complexity that is close to the waking state and is very different from other agents such as propofol or xenon in that respect (Sarasso et al., 2015). Indeed, ketamine increases the randomness of the EEG signal (Wang et al., 2017). Reduced alpha power in the precuneus and temporal-parietal junction, both regions involved in multisensory integration and body representation, has been proposed as a mechanism for the ketamine-induced dissociative altered consciousness state and disconnected consciousness (Darracq et al., 2018a; Vlisides et al., 2018). Effects of ketamine on other functional systems have also been evidenced, such as its long-term effect on the interactions between the default-mode network and other networks involved in depression pathophysiology (Li et al., 2018), the restoration of abnormal connectivity observed in depressed patients (Scheidegger et al., 2012; Vutskits, 2018), and a transient effect on a network involved in working memory (Driesen et al., 2013a).

### Dexmedetomidine

At first glance, the brain functional connectivity profile of the alteration of consciousness induced by the α_2_-adrenoceptor agonist dexmedetomidine appears similar to the one induced by physiological sleep and propofol, with a reduced within-network and thalamic connectivity in the higher-order consciousness networks, and a preservation of lower-order sensory networks (Guldenmund et al., 2017). However, dexmedetomidine better preserves functional connectivity between the thalamus, the anterior cingulate cortex, and the mesopontine area than sleep or propofol sedation (Guldenmund et al., 2017), all regions that are parts of the salience network. This may be in relation with the ability of dexmedetomidine to induce a state where the subject retains the capacity of rapidly recovering oriented responsiveness to external stimulation. From a topological point of view, dexmedetomidine reduces local and global large-scale network efficiency and connectivity strength, without impairing node degree (Hashmi et al., 2017).

### Benzodiazepines

Similarly to propofol, benzodiazepines, which are potent GABA receptor ligands, are known to preserve functional connectivity in lower-order sensory networks despite reduced direct auditory cortex activation by sounds (Frolich et al., 2017), but not in the higher-order consciousness networks (Greicius et al., 2008; Liang et al., 2015). They also reduce the duration and propagation of evoked TMS cortical responses (Ferrarelli et al., 2010). A long term and chronic administration of diazepam increase functional connectivity in areas of emotional processing (Pflanz et al., 2015).

### Xenon

Brain functional studies using the noble gas xenon are still scarce. This agent has anti-NMDA properties and interferes with potassium channels (Bonhomme et al., 2011). It reduces the activity of specific brain regions including the orbito- and mesiofrontal cortex, cingulate gyrus, thalamus, hippocampus and bilateral cerebellum (Rex et al., 2008). Similarly to propofol, it slows down and smooths the temporal profile of the EEG signal, and slides the brain state away from criticality (Colombo et al., 2019). TMS-evoked cortical responses under xenon anesthesia correspond to high amplitude slow waves with low complexity as compared to the wake state (Sarasso et al., 2015).

## Emerging Issues

**Figure 2:**
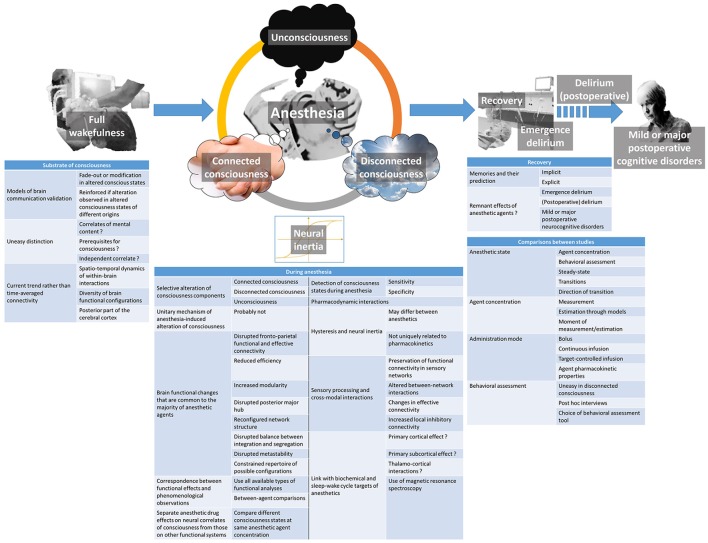
Summary of the currently emerging issues regarding the brain effects of anesthetic agents and their relationship with the postulated neural mechanisms of consciousness.

Despite huge progress in unraveling the modalities of within-brain interactions, their implications in consciousness generation, and defining concepts that were not even imagined a decade ago, each discovery in the domain of consciousness physiology and brain effects of anesthetic agents leads to new questions. The ones of relevance for the understanding of anesthetic action, and whose resolution will allow making progress in the understanding of consciousness itself, are discussed hereafter (Figure 2).

### Substrate of Consciousness and Its Alteration

Different models of within-brain communication were proposed as being witnesses of conscious processing by the brain following their fade-out or modification during altered states of consciousness. The strength of certainty when proposing a model is always reinforced when similar modifications are observed during alterations of consciousness of different origins (Lee et al., 2018). However, making the distinction between the effective neural correlates of consciousness (those responsible for the mental content experience), the elements that may support consciousness but are not mandatory (such as the sub-cortical arousal systems), and the neural systems whose alteration is simply a consequence or independent correlate of unconsciousness is not always easy (Boly et al., 2017; Mashour and Hudetz, 2018). Some authors distinguish content-specific neural correlates of consciousness (related to a specific mental content but not necessarily involved in another one), and full neural correlates of consciousness (mandatory for a mental content to be present; Boly et al., 2017). Based on lesion data, electrical or magnetic stimulation data, and functional brain imaging data, they argue that both content-specific and full neural correlates of consciousness are mainly located in the posterior part of the brain, encompassing the parietal, occipital, and lateral temporal lobes (Boly et al., 2017). This view is opposed to the previously prevailing one that was attributing a major role to the prefrontal cortex in this respect. It seems, however, that the prefrontal cortex is important at regulating the level of consciousness, through its privileged reciprocal interactions with subcortical arousal systems, in addition to attention, working memory, verbal and motor report processes (Pal et al., 2018). Information integration would occur through between-brain regions correlation in activity or functional connectivity (Koch et al., 2016), while information differentiation would be linked to the spatio-temporal dynamics of within-brain interactions and the diversity of brain functional configurations (Mashour and Hudetz, 2018; Demertzi et al., 2019). During anesthesia, however, one may not consider consciousness as a whole, insofar as specific aspects of it can be altered separately.

Further, matching up the evidence coming from different study paradigms and consciousness alterations of different origins, and finding parallelisms in the modification of functional witnesses of within-brain interaction models according to a specified component of consciousness will certainly lead to progress in characterizing its neural correlates with precision. By precisely and reversibly modulating consciousness, anesthesia will continue to play a major role in this progress. However, when analyzing anesthesia studies, researchers should pay attention to not mixing up the specific anesthetic drug effects on these neural correlates and their non-specific effects on neural functional assemblies that sustain other brain functions. Some study designs may help to overcome the difficulty when they involve a modulation of the consciousness state without changing the anesthetic agent concentration. As suggested by Scheinin et al. (2018), this can be achieved using external stimulation or an additional pharmacological agent that promotes cortical arousal through the strengthening of some subcortical arousal neurotransmission systems. External stimulation is more likely to provoke a change in the consciousness state when sedation is provided by an α_2_-adrenoceptor agonist like dexmedetomidine, but this can also occur under propofol sedation (Scheinin et al., 2018), or during routine general anesthesia when noxious stimulation elicits an episode of connected consciousness (Sanders et al., 2017). Similarly, physostigmine may change the level of arousal through an increase in cholinergic neurotransmission (Xie et al., 2011). When comparing the different brain states obtained at the same anesthetic agent concentration, it is important to precisely define their phenomenological characteristics, in order to pertinently correlate the functional changes with the behavioral ones.

### Specific Consciousness States of Anesthesia and the Associated Memory Processes

Dreaming and/or connectedness can occur frequently in apparently and behaviorally unresponsive subjects during anesthesia (Radek et al., 2018). The ability to sensitively and specifically distinguish between the different possible consciousness states of anesthesia, namely unconsciousness, disconnected consciousness, and connected consciousness, in addition to the ability of predicting implicit or explicit recall would be ideal. One must admit that this is currently not the case with classical EEG analyses and commercially available depth of anesthesia monitors, as shown in a study by Gaskell et al. (2017). In this isolated forearm technique study, only the alpha-slow wave phase-amplitude coupling in the EEG was able to discriminate between patients with connected consciousness and those not responding to command. The first step before achieving such ability is to develop an understanding of the involved mechanisms, so as to define recordable correlates. Up to now, the memory processes of anesthesia have been poorly studied from a functional point of view. For example, a low dose of sevoflurane blocks emotional memory by reducing functional connectivity between the amygdala and the hippocampus (Alkire et al., 2008), and ketamine reversibly interferes with the circuits of working memory (Driesen et al., 2013a). Brief episodes of connected consciousness following intense noxious events during anesthesia are rarely—if not never—followed by explicit recall, but could potentially lead to implicit memories (Sanders et al., 2017). The proportion of true unexpected awareness episodes with explicit recall over the total number of detected and undetected connected consciousness episodes is currently not known. Nevertheless, very few elements are available to characterize the functional status of the brain at the very moment of connected consciousness, and its ability to initiate and consolidate memories at that time. Again, hints of mechanistic explanations to the anesthesia-induced consciousness states begin to be obtained, essentially by comparing altered consciousness states of different origins, but with the same phenomenological characteristics, and by correlating the observations with implicit and explicit memory data. Hence, when simultaneously measuring the effects of different anesthetic agents on brain activity, it was shown that a steeper decay-rate of the resting EEG power spectral density was characteristic of the absence of mental content in unresponsive subjects under propofol or xenon anesthesia. At the same time, a power spectral density decay similar to wakefulness was indicative of a mental content in unresponsive subjects under ketamine anesthesia (Colombo et al., 2019). TMS-evoked alpha power was found to be reduced during disconnected consciousness (dreaming) as compared to wakefulness, whenever it occurs during ketamine anesthesia or rapid-eye-movement sleep (Darracq et al., 2018a). Decreased TMS-evoked gamma power was observed in unconsciousness states induced by propofol anesthesia or non-rapid eye movement sleep (Darracq et al., 2018a) as compared to wakefulness and the other states. Slow-wave EEG activity saturation also seems to be pathognomonic of disconnectedness from the environment (Warnaby et al., 2017). All mechanistic information obtainable through this approach has not been gathered yet, but will certainly be in the future.

### Sensory Processing and Cross-modal Interactions During Anesthesia

Within the scope of defining the amount of environmental information that reaches the brain, and the extent of its processing during the different anesthetic consciousness states, the study of sensory processing and cross-modal interactions is important. An intriguing recurrent finding of time-averaged connectivity studies is the preservation of functional connectivity within sensory networks, including thalamo-cortical connectivity, even during anesthesia-induced unresponsive states (Boveroux et al., 2010; Guldenmund et al., 2013; Bonhomme et al., 2016). However, sensory information handling by the brain appears to be altered during anesthesia through anomalies in between-network sensory modality crosstalk (Boveroux et al., 2010), changes in effective connectivity (Gómez et al., 2013), and increases in local inhibitory connectivity (Gómez et al., 2013). The use of more dynamic analysis paradigms and the study of evoked responses (Nourski et al., 2018) will certainly bring new information in this domain soon.

### Mixing Anesthetic Agents

As stated above, general anesthesia is rarely provided to patients using a single agent. Commonly, anesthetic agents with hypnotic properties are at least combined with opioids. It has been known for a long time that pharmacodynamic interactions between anesthetic agents exist, manifesting notably on routinely used processed EEG indexes of anesthetic depth (Bouillon et al., 2004). Very few information is currently available regarding those interactions at the level of within-brain communication, as well as information generation and handling by the brain. This topic merits further investigations using the existing functional brain imaging techniques.

### Direction-Dependent Mechanistic Differences in Anesthetic State Transitions

A phenomenon of hysteresis, sometimes referred to as neural inertia (Warnaby et al., 2017), occurs during anesthetic state transitions, meaning that the sequence of functional changes is distinct during forward and reverse transition from one consciousness state to the other. Although neural inertia typically refers to the pharmacokinetics of anesthetic agents, the observed differences are not uniquely related to them (Lee and Mashour, 2018b). This phenomenon may differ between anesthetic agents and is larger with the more potent ones (Kuizenga et al., 2018). Several EEG-based pharmacodynamic measures have been used to track the anesthesia recovery process (Purdon et al., 2013), and a phenomenon of hysteresis has been observed for the power spectrum, connectivity measures, structure and strength of networks (Kim et al., 2018), sensory-evoked EEG responses (Lewis et al., 2018), and slow-wave EEG activity saturation (Warnaby et al., 2017). The proposed involved mechanisms remain elusive. Specific study paradigms that precisely look at the dynamics of the transition loops and using different functional brain imaging modalities will help to elucidate them.

### Post-operative Remnant Effects of Anesthetic Agents

The effects of anesthetic agents on the brain do not cease when the syringe pump or the vaporizer are turned off. A series of perioperative neurocognitive disorders (NCD’s) may be encountered in patients beneficiating from surgery. The taxonomy of perioperative NCD’s has recently been redefined by a group of experts (Evered et al., 2018). When considering the NCD’s that may have a link with anesthetic agents themselves, the following entities can be described. Immediately after anesthesia and the recovery of consciousness, cognition may remain altered for a limited period of time, a condition called emergence delirium. New NCD’s that appear within the usually expected recovery period of 30 post-operative days are qualified as delirium or delayed neurocognitive recovery. The qualifier post-operative is added to delirium when new and persisting before discharge from the hospital, but not when appearing after discharge. After 30 days up to 12 months, persisting NCD’s are named mild or major post-operative NCD’s. They correspond to the previously used term of post-operative cognitive disorders (POCD). These entities may present over a wide variety of symptoms, ranging from an agitated and restless disorientation in time and space, to slight memory or other cognitive deficits (Mason et al., 2010). Very few information exists regarding their neural correlates and the underlying alterations in brain function. As compared to normal immediate post-operative recovery where functional and directed back-to-front connectivity is slowed down (Lee et al., 2013; van Dellen et al., 2014), hypoactive delirium is marked out by a less integrated network topology (Numan et al., 2017). Additional further studies are needed to fully understand the diversity of these cognitive disorders.

### Unitary Mechanism of Anesthesia-Induced Alteration of Consciousness

The question of a unitary mechanism of anesthesia-induced unconsciousness is a falsehood. Anesthetic agents produce different altered states of consciousness as a function of agent type and dose. This cannot occur through a common pathway. Several brain functional changes seem to be shared by the majority of anesthetic agents with hypnotic properties, including disrupted fronto-parietal functional and effective connectivity, reduced efficiency, increased modularity, disrupted posterior major hub, reconfigured network structure, disrupted balance between integration and segregation, disrupted metastability, and constrained repertoire of possible configurations (Lee and Mashour, 2018a). Among the observed functional effects, a good approach would be to establish a correspondence between them and phenomenological observations such as response to command when using the isolated forearm technique, *post hoc* report of dreaming with details, explicit recall of connected consciousness, standardized questionnaires for the detection of awareness, or specific paradigms for the detection of implicit memories. By submitting agent-related data to all available types of functional analyses, and making between-agent comparisons while taking into account the phenomenological characteristics of the consciousness states, substantial progress will be made in the understanding of the mechanism of each agent, and the functional correlate of consciousness components.

### Link Between Network Effects, Sleep-Wake Regulation, and Biochemical Targets

Anesthetic agents with hypnotic properties pertain to highly variable chemical families and have differing biochemical targets. They have also been shown to interfere with several subcortical neurotransmission systems that are involved in the regulation of the sleep-wake cycle (Bonhomme et al., 2011), although anesthesia is clearly distinct from physiological sleep (Akeju and Brown, 2017). As a corollary, the crux of the matter remains to link those observations with the evidenced network effects. Some postulate a primary cortical effect, particularly for those agents mainly promoting the inhibitory GABA neurotransmission (Brown et al., 2011). Others favor a primary subcortical site of action and cortical dysfunction as mainly originating from thalamo-cortical interaction changes (Hutt et al., 2018). Dexmedetomidine, whose main target is the subcortical noradrenergic system, is an exception (Nelson et al., 2003). Nevertheless, each agent probably has its own sequential scheme of action, primarily cortical or subcortical, and we are still far from having drawn the complete picture for each of them. Magnetic resonance spectroscopy studies looking at *in vivo* neurotransmission will probably offer new insights soon (Abdallah et al., 2018).

### Dose, Administration Mode, Anesthetic State, and Comparisons

The published studies in the domain of anesthetic drug effects on brain function considerably vary in their design, sometimes rendering the comparison between attained sedation level (when comparing different anesthetic agents), anesthetic doses, and studied anesthetic states hazardous. The phenomenological assessment of behavioral changes can sometimes be uneasy to perform (like during disconnected consciousness) or must be made through *post hoc* interviews. Behavioral assessment tools may also differ between studies. In addition, the route and mode of administration, which depend on the nature (intravenous or inhaled) and pharmacokinetic properties of the anesthetic agent, may introduce confounders. For example, a single intravenous bolus produces a continuously evolving alteration of consciousness, while a computer-controlled target infusion allows steady-state conditions of recording. Looking at steady-state anesthetic conditions does not provide the same information as looking at transitions from one state to the other. As mentioned above, the direction of the transition is also important. Precisely measuring or estimating the attained plasma or effect-site concentration of the anesthetic agent at the moment of recording is not always possible or not always performed. Indeed, due to inter-individual variability, the same anesthetic agent concentration may produce a different effect from one subject to the other, and some authors prefer defining the studied anesthetic state behaviorally rather than in terms of attained concentration. As a consequence, and given the high variability in study protocols regarding dose, mode of administration, and achieved anesthetic states, it is not easy to have a clear idea of the exact dose-response relationships, either in terms of brain function modifications and correspondences with phenomenological observations. A continuum in the observed effects from light to very deep sedation is not necessarily a reality, these relationships may be different from one anesthetic agent to the other, and not all possible levels of sedation have been studied for all anesthetic agents. All types of knowledge derived from the published studies are informative, but caution should be paid to not drawing erroneous conclusions when comparing them.

## Conclusion

The relationship between general anesthesia, brain function, and consciousness mechanisms is complex. As a matter of fact, anesthetic agents do not blunt out brain function globally but exert specific and dose-dependent effects on brain systems that sustain internal consciousness and perception of the environment. Each agent has its own mechanism of action, and dose-dependently induces distinct phenomenological altered consciousness states. Answering the questions that have recently emerged following recent discoveries on anesthetic brain effects will probably permit new insights into the specific diagnoses of anesthesia-induced altered states of consciousness, and into the understanding of the different aspects of consciousness itself. In that respect, general anesthesia can be considered as a flexible probe to explore consciousness.

## Author Contributions

VB and OG wrote the first draft of this article, the other authors equally participated in the revision and editing of the manuscript.

## Conflict of Interest Statement

During the past 5 years, VB has received research grant from Orion Pharma and honoraria for consultancy from Medtronic. The remaining authors declare that the research was conducted in the absence of any commercial or financial relationships that could be construed as a potential conflict of interest.
